# Retinal Detachment due to CrossFit Training Injury

**DOI:** 10.1155/2013/189837

**Published:** 2013-09-11

**Authors:** Stephanie A. Joondeph, Brian C. Joondeph

**Affiliations:** Colorado Retina Associates, PC 8101 E Lowry Boulevard, Suite 210, Denver, CO 80230, USA

## Abstract

The purpose of this paper is to describe a traumatic retinal detachment occurring as a result of CrossFit training using an elastic exercise band. The patient sustained an ocular injury from an elastic band during CrossFit training, resulting in a giant retinal dialysis and retinal detachment, which were successfully repaired. Trainers and athletes need to be aware of the potential for ocular injury from elastic exercise bands and take appropriate precautions.

## 1. Introduction

 CrossFit is a new strength and conditioning program with short but intense daily workouts. These workouts include strength and resistance training with elastic resistance bands. We wish to report a case of a retinal detachment occurring following elastic band breakage during a CrossFit workout.

## 2. Design and Methods

 This is a single patient case report.

## 3. Findings

 A twenty-five-year old male presented with an inferior scotoma in his right eye for two days. Ten days earlier an elastic resistance band snapped and hit his right eye. He was engaged in a CrossFit workout doing pull ups with an elastic band tied around his waist and secured to the pull up bar thus partially supporting his weight. His ocular examination revealed visual acuity of 20/30 OD, 20/20 OS. His anterior segments were normal. Right fundus exam revealed a three-clock hour superotemporal retinal dialysis, with vitreous base avulsion and localized retinal detachment not involving the macula. One day later, he underwent scleral buckling surgery of his right eye using cryopexy, an encircling band, subretinal fluid drainage, and gas bubble. His retina was successfully reattached, and his vision was 20/25 OD after four months of followup.

## 4. Discussion

**Figure 1:**
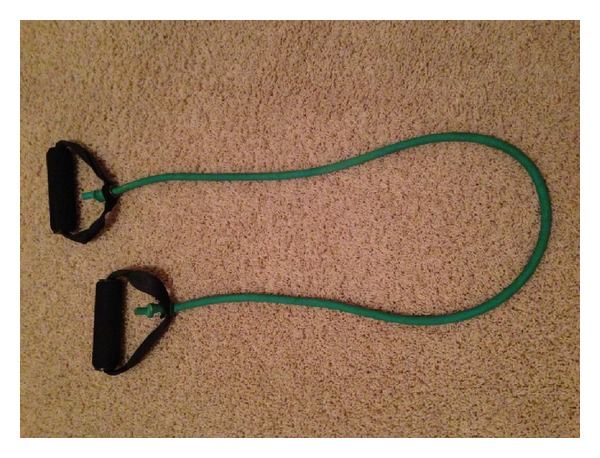
Elastic exercise band.

 This patient had a traumatic giant retinal dialysis and retinal detachment as a consequence of elastic resistance band breakage during CrossFit training. To our knowledge, this type of injury has not been reported. Elastic bands are being used increasingly as an exercise accessory to provide resistance, not only in CrossFit, but also in other popular exercise programs, such as P90X. Some elastic bands consist of latex strips of variable lengths and resistance while others are elastic tubes with soft grip handles on the ends (Figure 1). With repeated use, elastic bands can weaken and eventually break. When it is under tension, a broken elastic band can snap into the eye causing injuries such as hyphema, cataract, or retinal detachment. Dialyses and giant retinal tears are responsible for 69 percent of traumatic retinal detachments [1].

 Elastic bungee cords have been reported to cause a variety of ocular injuries [2, 3]. Elastic resistance bands might appear to be safer since they do not have metallic hooks on their ends as bungee cords do, although some have soft handles. Nevertheless, an elastic band, under high tension, can snap causing a high velocity missile impact with the eye, causing similar injuries to a bungee cord. Exercise participants and trainers should be aware of the potential for this type of injury. Precautions include the use of protective eyewear and regular replacement of worn elastic bands. Examining bands before and after use, looking for nicks, punctures, or tears, and discarding worn bands may prevent these injuries.
